# *rac*-7-Methyl-3-[(7-methyl-4-oxo­chro­man-3-yl)meth­yl]-4*H*-chromen-4-one

**DOI:** 10.1107/S1600536813009422

**Published:** 2013-04-20

**Authors:** M Somasundaram, A. Rajendiran, K.K. Balasubramanian, K. Krishnasamy, S. Kabilan

**Affiliations:** a Department of Chemistry, Annamalai University, Annamalai Nagar, Chidambaram, India; b Shasun Reaearch Centre, 27 Vandaloor Kelambakkam Road, Keezhakottaiyur, Meelakottaiyur Post, Chennai, India

## Abstract

In the racemic title compound, C_21_H_18_O_4_, the chromone ring is essentially planar [maximum deviation from the least-squares plane = 0.026 (3) Å], with a dihedral angle of 78.18 (12)° between the benzene rings of the chromanone and chromenone moieties. In the crystal, there are weak π–π stacking inter­actions [minimum ring centroid separation = 3.9286 (17) Å].

## Related literature

For backgound to bis-chromanones, see: Dean & Murray (1975 ▶); Santhosh & Balasubramanian (1991 ▶); Panja *et al.* (2009 ▶). For related structures, see: Ambartsumyan *et al.* (2012 ▶); Nyburg *et al.* (1986 ▶); Li *et al.* (2010 ▶).
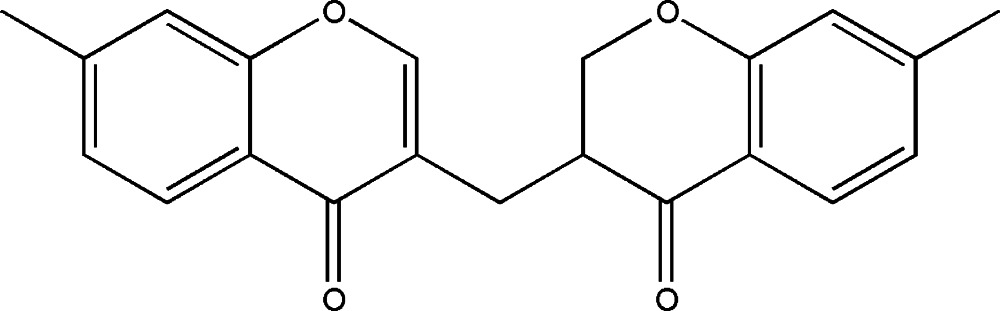


## Experimental

### 

#### Crystal data

C_21_H_18_O_4_
*M* *_r_* = 334.35
Monoclinic, 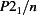

*a* = 10.664 (2) Å
*b* = 6.6428 (13) Å
*c* = 23.754 (5) Å
β = 91.11 (3)°
*V* = 1682.4 (6) Å^3^
*Z* = 4
Mo *K*α radiation
μ = 0.09 mm^−1^
*T* = 293 K
0.44 × 0.22 × 0.22 mm

#### Data collection

Bruker SMART CCD diffractometer
Absorption correction: multi-scan (*SADABS*; Bruker, 2007 ▶) *T*_min_ = 0.961, *T*_max_ = 0.980
11780 measured reflections
4032 independent reflections
1982 reflections with *I* > 2σ(*I*)
*R*_int_ = 0.029

#### Refinement

*R*[*F* ^2^ > 2σ(*F* ^2^)] = 0.059
*wR*(*F* ^2^) = 0.180
*S* = 1.02
4032 reflections
229 parameters
H-atom parameters constrained
Δρ_max_ = 0.43 e Å^−3^
Δρ_min_ = −0.30 e Å^−3^

### 

Data collection: *SMART* (Bruker, 2007 ▶); cell refinement: *SAINT* (Bruker, 2007 ▶); data reduction: *SAINT*; program(s) used to solve structure: *SHELXS97* (Sheldrick, 2008 ▶); program(s) used to refine structure: *SHELXL97* (Sheldrick, 2008 ▶); molecular graphics: *SHELXTL* (Sheldrick, 2008 ▶); software used to prepare material for publication: *SHELXTL*.

## Supplementary Material

Crystal structure: contains datablock(s) I, global. DOI: 10.1107/S1600536813009422/zs2250sup1.cif

Structure factors: contains datablock(s) I. DOI: 10.1107/S1600536813009422/zs2250Isup2.hkl

Supplementary material file. DOI: 10.1107/S1600536813009422/zs2250Isup3.cml

Additional supplementary materials:  crystallographic information; 3D view; checkCIF report

**Fig. 1.**
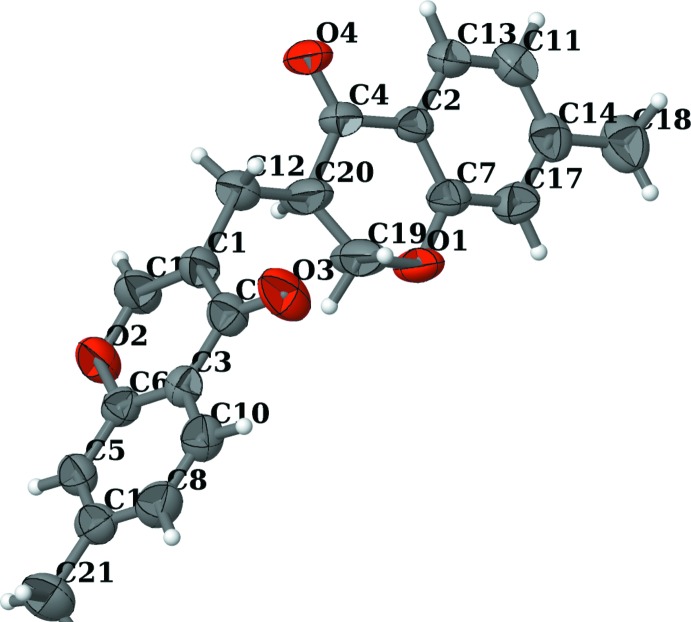
The molecular conformation and atom numbering scheme for the title compound, showing 50% probability displacement ellipsoids.

Enhanced figure: interactive version of Fig. 1

## supplementary crystallographic information

### Comment

The chromanone moiety forms an important component in pharmacophores in a number of biologically active molecules of synthetic as well as natural origin. Bis-chromanones bridged by a methylene group at C3 of the ring are considered to be a biologically important class of molecules (Santhosh & Balasubramanian, 1991; Panja, *et al.*, 2009). Herein we report the structure of the racemic title compound, C_21_H_18_O_4_, in which the dihedral angle between the phenyl rings of the two chromanone moieties is 78.18 (12)° (Fig. 1). The chromone ring is essentially planar [maximum deviation from the l.s. plane = 0.026 (3) Å (C15)], while in the chromanone ring the maximum deviation is 0.206 (4) Å (C19)]. In the chromanone ring system, the C19—C20 bond length is short [1.380 (5)]. The torsion angles about the central methylene carbon C12 (C15—C1—C12—C20 and C19—C20—C12—C1) are -93.91 (3) and -37.48 (4)°, respectively. The angle subtended at C12 by the C—C bonds (C1—C12—C20) is 114.87 (2)°. The olefinic bond length [C1—C15 = 1.324 (4) Å] is close to the values found in known chromanone systems (Ambartsumyan *et al.*, 2012). Some examples of bis-chromanone structures are known (Dean & Murray, 1975). In the crystal, there are weak π···π stacking interactions [minimum ring centroid separation = 3.9286 (17) Å].

### Experimental

In a dry single-neck round-bottom flask, 4-chloro-3-formyl chromene (1 mol) and sodium acetate (1.1 mol) was taken, and DMF (5 vol) was added. The reaction mixture was stirred at 70 – 80 °C for 7–8 h. The progress of the reaction was monitored by TLC. After completion of the reaction, the mixture was cooled to room temperature and then quenched with water, extracted with ethyl acetate and concentrated under reduced pressure, giving the crude bis-chromanone product. This product was purified on silica gel using ethyl acetate–hexane solvent, giving the pure title compound.

### Crystal data

**Table:**

C_21_H_18_O_4_	*F*(000) = 704
*M**_r_* = 334.35	*D*_x_ = 1.320 Mg m^−^^3^
Monoclinic, *P*2_1_/*n*	Mo *K*α radiation, λ = 0.71073 Å
Hall symbol: -P 2yn	Cell parameters from 2970 reflections
*a* = 10.664 (2) Å	θ = 2.5–28.2°
*b* = 6.6428 (13) Å	µ = 0.09 mm^−^^1^
*c* = 23.754 (5) Å	*T* = 293 K
β = 91.11 (3)°	Crystal, yellow
*V* = 1682.4 (6) Å^3^	0.44 × 0.22 × 0.22 mm
*Z* = 4	

### Data collection

**Table:**

Bruker SMART CCD diffractometer	4032 independent reflections
Radiation source: fine-focus sealed tube	1982 reflections with *I* > 2σ(*I*)
Graphite monochromator	*R*_int_ = 0.029
φ and ω scans	θ_max_ = 28.3°, θ_min_ = 3.2°
Absorption correction: multi-scan (*SADABS*; Bruker, 2007)	*h* = −13→12
*T*_min_ = 0.961, *T*_max_ = 0.980	*k* = −8→8
11780 measured reflections	*l* = −29→31

### Refinement

**Table:**

Refinement on *F*^2^	Primary atom site location: structure-invariant direct methods
Least-squares matrix: full	Secondary atom site location: difference Fourier map
*R*[*F*^2^ > 2σ(*F*^2^)] = 0.059	Hydrogen site location: inferred from neighbouring sites
*wR*(*F*^2^) = 0.180	H-atom parameters constrained
*S* = 1.02	*w* = 1/[σ^2^(*F*_o_^2^) + (0.0748*P*)^2^ + 0.4282*P*] where *P* = (*F*_o_^2^ + 2*F*_c_^2^)/3
4032 reflections	(Δ/σ)_max_ < 0.001
229 parameters	Δρ_max_ = 0.43 e Å^−^^3^
0 restraints	Δρ_min_ = −0.30 e Å^−^^3^

### Special details

**Table:**

Geometry. All e.s.d.'s (except the e.s.d. in the dihedral angle between two l.s. planes) are estimated using the full covariance matrix. The cell e.s.d.'s are taken into account individually in the estimation of e.s.d.'s in distances, angles and torsion angles; correlations between e.s.d.'s in cell parameters are only used when they are defined by crystal symmetry. An approximate (isotropic) treatment of cell e.s.d.'s is used for estimating e.s.d.'s involving l.s. planes.
Refinement. Refinement of *F*^2^ against ALL reflections. The weighted *R*-factor *wR* and goodness of fit *S* are based on *F*^2^, conventional *R*-factors *R* are based on *F*, with *F* set to zero for negative *F*^2^. The threshold expression of *F*^2^ > σ(*F*^2^) is used only for calculating *R*-factors(gt) *etc*. and is not relevant to the choice of reflections for refinement. *R*-factors based on *F*^2^ are statistically about twice as large as those based on *F*, and *R*- factors based on ALL data will be even larger.

### Fractional atomic coordinates and isotropic or equivalent isotropic displacement parameters (Å^2^)

**Table:**

	*x*	*y*	*z*	*U*_iso_*/*U*_eq_	
O2	−0.12544 (16)	−0.0887 (3)	0.10824 (8)	0.0673 (5)	
O1	0.34094 (18)	0.1176 (2)	−0.01163 (8)	0.0682 (5)	
C1	0.0420 (2)	0.1514 (3)	0.10862 (11)	0.0573 (7)	
C2	0.3259 (2)	0.4731 (3)	−0.03163 (10)	0.0472 (6)	
O4	0.18080 (18)	0.6629 (2)	0.01958 (8)	0.0729 (6)	
C3	0.0456 (2)	−0.1335 (3)	0.17464 (9)	0.0471 (6)	
C4	0.2280 (2)	0.4997 (3)	0.00924 (10)	0.0537 (6)	
C5	−0.1317 (2)	−0.3631 (3)	0.16993 (11)	0.0566 (7)	
H5	−0.2075	−0.4003	0.1529	0.068*	
C6	−0.0679 (2)	−0.1937 (3)	0.15148 (10)	0.0493 (6)	
C7	0.3769 (2)	0.2828 (3)	−0.04092 (10)	0.0533 (6)	
C8	0.0330 (3)	−0.4157 (4)	0.23729 (12)	0.0684 (8)	
H8	0.0676	−0.4909	0.2667	0.082*	
O3	0.2109 (2)	0.1038 (3)	0.17275 (9)	0.0836 (6)	
C9	0.1091 (2)	0.0457 (4)	0.15384 (10)	0.0550 (6)	
C10	0.0960 (2)	−0.2503 (4)	0.21857 (10)	0.0604 (7)	
H10	0.1726	−0.2152	0.2351	0.072*	
C11	0.4571 (3)	0.6020 (4)	−0.10497 (12)	0.0634 (7)	
H11	0.4826	0.7100	−0.1269	0.076*	
C12	0.0952 (3)	0.3414 (3)	0.08411 (11)	0.0606 (7)	
H12A	0.0263	0.4252	0.0708	0.073*	
H12B	0.1391	0.4145	0.1139	0.073*	
C13	0.3700 (2)	0.6324 (3)	−0.06455 (11)	0.0581 (7)	
H13	0.3392	0.7615	−0.0587	0.070*	
C14	0.5085 (2)	0.4123 (4)	−0.11405 (11)	0.0631 (7)	
C15	−0.0676 (3)	0.0812 (4)	0.09029 (12)	0.0684 (8)	
H15	−0.1090	0.1553	0.0624	0.082*	
C16	−0.0817 (3)	−0.4749 (4)	0.21350 (11)	0.0605 (7)	
C17	0.4669 (3)	0.2547 (4)	−0.08142 (12)	0.0652 (7)	
H17	0.5001	0.1268	−0.0868	0.078*	
C18	0.6082 (3)	0.3821 (5)	−0.15727 (14)	0.0897 (10)	
H18A	0.5698	0.3752	−0.1941	0.135*	
H18B	0.6525	0.2591	−0.1495	0.135*	
H18C	0.6660	0.4930	−0.1558	0.135*	
C19	0.2677 (4)	0.1520 (4)	0.03518 (17)	0.0991 (12)	
H19	0.2205	0.0297	0.0418	0.119*	
H1	0.3247	0.1687	0.0672	0.62 (11)*	
C20	0.1836 (3)	0.3097 (4)	0.03647 (15)	0.0846 (10)	
H20	0.1236	0.2640	0.0074	0.102*	
C21	−0.1514 (3)	−0.6556 (5)	0.23528 (16)	0.0968 (11)	
H21A	−0.1730	−0.6333	0.2738	0.145*	
H21B	−0.0990	−0.7727	0.2328	0.145*	
H21C	−0.2264	−0.6758	0.2130	0.145*	

### Atomic displacement parameters (Å^2^)

**Table:**

	*U*^11^	*U*^22^	*U*^33^	*U*^12^	*U*^13^	*U*^23^
O2	0.0557 (11)	0.0714 (11)	0.0742 (12)	−0.0134 (9)	−0.0163 (10)	0.0252 (10)
O1	0.0876 (14)	0.0363 (8)	0.0811 (13)	0.0084 (8)	0.0148 (11)	0.0026 (8)
C1	0.0505 (16)	0.0538 (13)	0.0673 (17)	−0.0022 (12)	−0.0023 (13)	0.0107 (12)
C2	0.0488 (14)	0.0389 (11)	0.0536 (14)	−0.0033 (10)	−0.0045 (12)	−0.0018 (10)
O4	0.0857 (14)	0.0385 (9)	0.0954 (14)	0.0122 (9)	0.0249 (11)	0.0127 (9)
C3	0.0463 (14)	0.0516 (12)	0.0434 (13)	0.0032 (10)	0.0013 (11)	0.0001 (10)
C4	0.0595 (16)	0.0373 (11)	0.0643 (16)	0.0021 (11)	0.0007 (13)	0.0061 (11)
C5	0.0528 (15)	0.0562 (14)	0.0610 (16)	−0.0059 (12)	0.0092 (13)	0.0018 (12)
C6	0.0500 (15)	0.0513 (12)	0.0466 (13)	0.0053 (11)	0.0026 (12)	0.0044 (11)
C7	0.0597 (16)	0.0413 (12)	0.0587 (15)	−0.0011 (11)	−0.0027 (13)	−0.0043 (11)
C8	0.0706 (19)	0.0727 (17)	0.0621 (17)	0.0132 (15)	0.0064 (15)	0.0242 (14)
O3	0.0722 (14)	0.0901 (14)	0.0873 (14)	−0.0270 (11)	−0.0280 (12)	0.0159 (11)
C9	0.0530 (16)	0.0582 (14)	0.0536 (15)	−0.0038 (12)	−0.0053 (13)	0.0008 (12)
C10	0.0554 (16)	0.0719 (16)	0.0537 (15)	0.0039 (13)	−0.0019 (13)	0.0093 (13)
C11	0.0646 (18)	0.0589 (15)	0.0666 (17)	−0.0138 (13)	0.0011 (15)	0.0009 (13)
C12	0.0640 (17)	0.0497 (13)	0.0683 (17)	−0.0035 (12)	0.0051 (14)	0.0063 (12)
C13	0.0581 (16)	0.0439 (12)	0.0723 (17)	−0.0029 (11)	0.0012 (14)	0.0009 (12)
C14	0.0568 (17)	0.0703 (17)	0.0622 (17)	−0.0111 (13)	−0.0008 (14)	−0.0147 (14)
C15	0.0621 (18)	0.0651 (16)	0.0775 (19)	−0.0048 (14)	−0.0101 (15)	0.0263 (14)
C16	0.0658 (18)	0.0550 (14)	0.0615 (16)	0.0045 (13)	0.0187 (14)	0.0104 (12)
C17	0.0693 (18)	0.0516 (14)	0.0746 (18)	0.0030 (13)	0.0015 (15)	−0.0140 (13)
C18	0.084 (2)	0.099 (2)	0.087 (2)	−0.0138 (18)	0.0216 (19)	−0.0229 (18)
C19	0.133 (3)	0.0430 (14)	0.124 (3)	0.0074 (17)	0.060 (3)	0.0136 (16)
C20	0.105 (2)	0.0431 (14)	0.107 (2)	0.0176 (14)	0.041 (2)	0.0204 (14)
C21	0.102 (3)	0.078 (2)	0.111 (3)	−0.0075 (18)	0.027 (2)	0.0322 (19)

### Geometric parameters (Å, º)

**Table:**

O2—C15	1.359 (3)	C10—H10	0.9300
O2—C6	1.376 (3)	C11—C13	1.364 (4)
O1—C7	1.359 (3)	C11—C14	1.393 (3)
O1—C19	1.390 (3)	C11—H11	0.9300
C1—C15	1.324 (3)	C12—C20	1.501 (4)
C1—C9	1.459 (4)	C12—H12A	0.9700
C1—C12	1.505 (3)	C12—H12B	0.9700
C2—C7	1.395 (3)	C13—H13	0.9300
C2—C13	1.402 (3)	C14—C17	1.381 (4)
C2—C4	1.451 (3)	C14—C18	1.505 (4)
O4—C4	1.222 (3)	C15—H15	0.9300
C3—C6	1.379 (3)	C16—C21	1.508 (4)
C3—C10	1.399 (3)	C17—H17	0.9300
C3—C9	1.460 (3)	C18—H18A	0.9600
C4—C20	1.499 (3)	C18—H18B	0.9600
C5—C16	1.373 (4)	C18—H18C	0.9600
C5—C6	1.390 (3)	C19—C20	1.380 (4)
C5—H5	0.9300	C19—H19	0.9700
C7—C17	1.385 (3)	C19—H1	0.9700
C8—C10	1.367 (4)	C20—H20	0.9800
C8—C16	1.394 (4)	C21—H21A	0.9600
C8—H8	0.9300	C21—H21B	0.9600
O3—C9	1.229 (3)	C21—H21C	0.9600
			
C15—O2—C6	117.2 (2)	H12A—C12—H12B	107.5
C7—O1—C19	116.44 (18)	C11—C13—C2	121.4 (2)
C15—C1—C9	119.3 (2)	C11—C13—H13	119.3
C15—C1—C12	120.3 (2)	C2—C13—H13	119.3
C9—C1—C12	120.3 (2)	C17—C14—C11	117.9 (2)
C7—C2—C13	117.4 (2)	C17—C14—C18	121.4 (3)
C7—C2—C4	120.3 (2)	C11—C14—C18	120.8 (3)
C13—C2—C4	122.3 (2)	C1—C15—O2	126.3 (2)
C6—C3—C10	117.3 (2)	C1—C15—H15	116.8
C6—C3—C9	120.7 (2)	O2—C15—H15	116.8
C10—C3—C9	122.1 (2)	C5—C16—C8	118.5 (2)
O4—C4—C2	123.2 (2)	C5—C16—C21	120.2 (3)
O4—C4—C20	121.7 (2)	C8—C16—C21	121.3 (3)
C2—C4—C20	115.07 (19)	C14—C17—C7	121.5 (2)
C16—C5—C6	119.4 (3)	C14—C17—H17	119.3
C16—C5—H5	120.3	C7—C17—H17	119.3
C6—C5—H5	120.3	C14—C18—H18A	109.5
O2—C6—C3	121.8 (2)	C14—C18—H18B	109.5
O2—C6—C5	115.6 (2)	H18A—C18—H18B	109.5
C3—C6—C5	122.7 (2)	C14—C18—H18C	109.5
O1—C7—C17	116.9 (2)	H18A—C18—H18C	109.5
O1—C7—C2	122.4 (2)	H18B—C18—H18C	109.5
C17—C7—C2	120.6 (2)	C20—C19—O1	121.2 (3)
C10—C8—C16	121.9 (2)	C20—C19—H19	107.0
C10—C8—H8	119.1	O1—C19—H19	107.0
C16—C8—H8	119.1	C20—C19—H1	107.0
O3—C9—C1	122.3 (2)	O1—C19—H1	107.0
O3—C9—C3	123.0 (2)	H19—C19—H1	106.8
C1—C9—C3	114.7 (2)	C19—C20—C4	114.7 (2)
C8—C10—C3	120.3 (3)	C19—C20—C12	122.7 (3)
C8—C10—H10	119.8	C4—C20—C12	114.6 (2)
C3—C10—H10	119.8	C19—C20—H20	99.5
C13—C11—C14	121.2 (2)	C4—C20—H20	99.5
C13—C11—H11	119.4	C12—C20—H20	99.5
C14—C11—H11	119.4	C16—C21—H21A	109.5
C20—C12—C1	114.9 (2)	C16—C21—H21B	109.5
C20—C12—H12A	108.5	H21A—C21—H21B	109.5
C1—C12—H12A	108.5	C16—C21—H21C	109.5
C20—C12—H12B	108.5	H21A—C21—H21C	109.5
C1—C12—H12B	108.5	H21B—C21—H21C	109.5
			
C19—O1—C7—C2	−11.4 (4)	C6—C3—C9—O3	−179.2 (2)
C19—O1—C7—C17	169.2 (3)	C6—C3—C9—C1	0.9 (3)
C7—O1—C19—C20	33.0 (4)	C10—C3—C9—O3	1.1 (4)
C15—O2—C6—C3	−2.0 (3)	C10—C3—C9—C1	−178.9 (2)
C15—O2—C6—C5	178.0 (2)	C6—C3—C10—C8	−0.5 (4)
C6—O2—C15—C1	3.3 (4)	C9—C3—C10—C8	179.2 (2)
C12—C1—C9—O3	−1.4 (4)	O4—C4—C20—C12	−10.6 (4)
C12—C1—C9—C3	178.6 (2)	O4—C4—C20—C19	−160.5 (3)
C15—C1—C9—O3	−179.8 (3)	C2—C4—C20—C12	172.1 (2)
C15—C1—C9—C3	0.2 (3)	C2—C4—C20—C19	22.2 (4)
C9—C1—C12—C20	87.7 (3)	C16—C5—C6—O2	−179.1 (2)
C15—C1—C12—C20	−93.9 (3)	C16—C5—C6—C3	1.0 (4)
C9—C1—C15—O2	−2.4 (4)	C6—C5—C16—C8	−0.9 (4)
C12—C1—C15—O2	179.3 (2)	C6—C5—C16—C21	178.2 (2)
C7—C2—C4—O4	179.2 (2)	O1—C7—C17—C14	179.0 (3)
C7—C2—C4—C20	−3.6 (3)	C2—C7—C17—C14	−0.4 (4)
C13—C2—C4—O4	−3.1 (4)	C16—C8—C10—C3	0.5 (4)
C13—C2—C4—C20	174.2 (2)	C10—C8—C16—C5	0.2 (4)
C4—C2—C7—O1	−2.0 (3)	C10—C8—C16—C21	−178.9 (3)
C4—C2—C7—C17	177.5 (2)	C14—C11—C13—C2	−1.9 (4)
C13—C2—C7—O1	−179.8 (2)	C13—C11—C14—C17	1.1 (4)
C13—C2—C7—C17	−0.4 (3)	C13—C11—C14—C18	−177.6 (3)
C4—C2—C13—C11	−176.3 (2)	C1—C12—C20—C4	175.3 (2)
C7—C2—C13—C11	1.5 (4)	C1—C12—C20—C19	−37.5 (4)
C9—C3—C6—O2	0.1 (3)	C11—C14—C17—C7	0.1 (4)
C9—C3—C6—C5	−180.0 (2)	C18—C14—C17—C7	178.8 (3)
C10—C3—C6—O2	179.8 (2)	O1—C19—C20—C4	−38.2 (5)
C10—C3—C6—C5	−0.2 (3)	O1—C19—C20—C12	174.7 (3)
